# Targeting Negative and Positive Immune Checkpoints with Monoclonal Antibodies in Therapy of Cancer

**DOI:** 10.3390/cancers11111756

**Published:** 2019-11-08

**Authors:** Katsiaryna Marhelava, Zofia Pilch, Malgorzata Bajor, Agnieszka Graczyk-Jarzynka, Radoslaw Zagozdzon

**Affiliations:** 1 Department of Clinical Immunology, Medical University of Warsaw, Nowogrodzka 59 Street, 02-006 Warsaw, Poland; k.marhelava@gmail.com (K.M.); malgorzata.bajor@wum.edu.pl (M.B.); 2 Postgraduate School of Molecular Medicine, Medical University of Warsaw, Trojdena 2a Street, 02-091 Warsaw, Poland; 3 Department of Immunology, Medical University of Warsaw, Nielubowicza 5 Street, 02-097 Warsaw, Poland; zofia.pilch@gmail.com (Z.P.); agnieszka.graczyk-jarzynka@wum.edu.pl (A.G.-J.); 4 Department of Immunology, Transplantology and Internal Diseases, Medical University of Warsaw, Nowogrodzka 59 Street, 02-006 Warsaw, Poland; 5 Institute of Biochemistry and Biophysics, Polish Academy of Sciences, Pawinskiego 5A Street, 02-106 Warsaw, Poland

**Keywords:** immune checkpoints, monoclonal antibodies, immunotherapy, tumor immunity, combination therapy

## Abstract

The immune checkpoints are regulatory molecules that maintain immune homeostasis in physiological conditions. By sending T cells a series of co-stimulatory or co-inhibitory signals via receptors, immune checkpoints can both protect healthy tissues from adaptive immune response and activate lymphocytes to remove pathogens effectively. However, due to their mode of action, suppressive immune checkpoints may serve as unwanted protection for cancer cells. To restore the functioning of the immune system and make the patient’s immune cells able to recognize and destroy tumors, monoclonal antibodies are broadly used in cancer immunotherapy to block the suppressive or to stimulate the positive immune checkpoints. In this review, we aim to present the current state of application of monoclonal antibodies in clinics, used either as single agents or in a combined treatment. We discuss the limitations of these therapies and possible problem-solving with combined treatment approaches involving both non-biological and biological agents. We also highlight the most promising strategies based on the use of monoclonal or bispecific antibodies targeted on immune checkpoints other than currently implemented in clinics.

## 1. Introduction

Traditional therapy of disseminated cancer is usually based on systemic treatment with several types of chemotherapy, including molecularly targeted small molecules. However, these kinds of treatment are often not effective enough to defeat cancer due to certain limitations [1]. Even if cancer cells are initially sensitive to standard antitumor modalities, and a rapid diminishment of tumor mass occurs, the eventual recurrence of a refractory cancer is frequent. Importantly, in standard systemic anticancer treatment, the intratumor diversity of cancer cell phenotypes can be a major cause for tumor resistance to the chemotherapeutic agent(s), as some phenotypes can be much more resistant than others to a given therapy. It is exactly the opposite in case of active immunotherapy targeting immune checkpoint molecules with monoclonal antibodies, as the more different the cancer cell is from the host, the stronger immunogenicity it presents. That makes active immunotherapy unique in its concept, as described in detail below. The therapeutic use of the immune checkpoint molecules in cancer treatment has been initiated by targeting the negative checkpoints, but recently growing attention has been paid to the role of positive immune checkpoints as targets for anticancer therapies. This review aims to present the comprehensive look at the current clinical status of both types of therapeutic approaches, and also to describe the most promising recent developments in the field.

## 2. Immune Checkpoints

Properly functioning human immune system shields our body from pathogens and developing malignancies [2]. However, when overactive and/or malfunctioning, the immune system poses a severe and potentially lethal threat to the human body. Therefore, the activation of the immune system, including its effector cells, such as T lymphocytes and natural killer (NK) cells, is closely monitored. In the case of T lymphocytes, recognition by T cell receptor (TCR) of the specific antigen presented by the major histocompatibility complex (MHC) molecules provides the first signal for activation [3]. This signal stimulates the lymphocytes only briefly and requires co-stimulation, for example from a CD28 molecule recognizing its ligands (CD80 or CD86) on antigen-presenting cell (APC), to introduce T lymphocyte into full activation [4]. This process is additionally regulated by various cytokines acting on the T lymphocyte [5], as well as by the so-called immune checkpoints [6]. 

**Figure 1:**
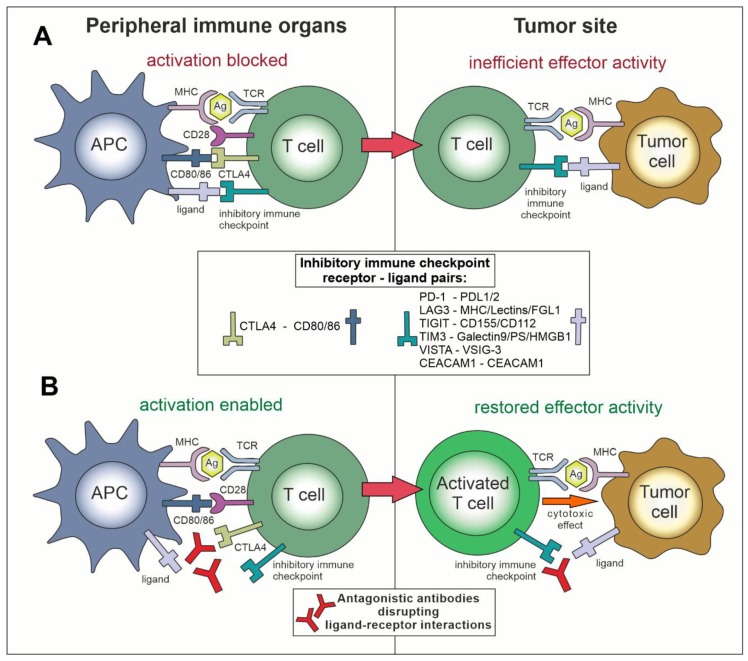
Targeting the negative immune checkpoints with monoclonal antibodies. (**A**) The mechanism of actions of negative immune checkpoints on the example of a T cell. CTLA-4 binds to CD80 and CD86 receptors on the antigen-presenting cells (APCs), outcompeting immuno-stimulatory CD28 binding, and thus dampening the T cell receptor (TCR) signaling. Additionally, some other ligands for inhibitory immune checkpoints on immune cells may be expressed on APCs, and most of them can be overexpressed on tumor cells and/or in tumor microenvironment. Interactions between inhibitory immune checkpoints and their ligands block the activation of immune effector cells and prevent their cytotoxic response towards tumor cells. (**B**) Application of antibodies antagonistic towards the negative immune checkpoint receptors and/or their ligands enables CD80/86 and CD28 immuno-stimulatory signaling and blocks immuno-inhibitory signaling through other negative immune checkpoints. Activated T cells can become capable of overcoming the regulatory mechanisms and elimination of the tumor cells.

**Figure 2:**
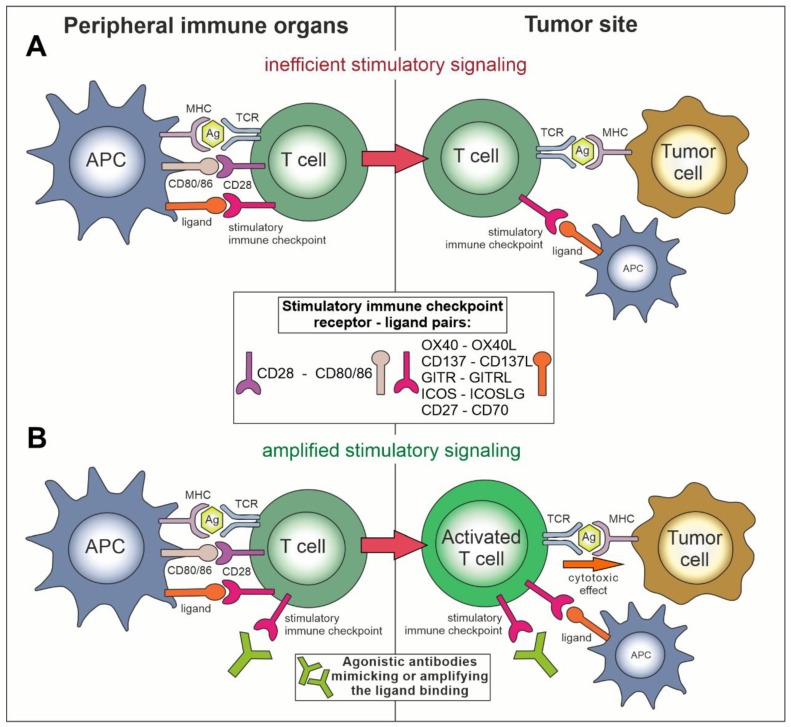
Targeting the positive immune checkpoints with monoclonal antibodies. (**A**) The mechanism of stimulation of a T cell effector function via positive immune checkpoints. The interaction of CD28 with its ligands, CD80 or CD86, follows TCR signaling and co-stimulates immune cell activation. Some of the other stimulatory immune checkpoints may also provide a co-stimulatory signal, but most of them start being expressed on already activated immune cells. In advanced cancer, this positive signaling is, however, often insufficient for eliminating the malignant cells. (**B**) Application of agonistic antibodies mimicking or amplifying binding of the ligands for stimulatory immune checkpoints increases effector activity of T cells towards tumor cells with prospective elimination of cancer cells.

Immune checkpoint receptors are membrane molecules, located mainly, but not exclusively, on T lymphocytes and NK cells, which, after recognizing appropriate ligands on the antigen-presenting cells (APC) or the target cells, can play a negative (inhibitory, Figure 1A) or positive (stimulatory, Figure 2A) role in the process of the lymphocyte activation. Immune checkpoints can propagate inhibitory or stimulatory signals via interactions of the checkpoint molecules, i.e., ligands with their cognate receptors located on target and effector cells, respectively (Table 1). Under normal physiological conditions, immune checkpoints are crucial players in maintaining immune homeostasis and preventing autoimmunity [7]. When compared to a motorized vehicle, the TCR-elicited signal would be starting the engine for the T cell, while the negative or positive checkpoints would function as the brake or accelerator, respectively. Setting up the right balance between them is responsible for moving the immune system response into the right direction—tolerance or attack, depending on circumstances. 

Checkpoint receptors, such as cytotoxic T-lymphocyte-associated protein 4 (CTLA-4), programmed cell death protein 1 (PD-1), lymphocyte-activation gene 3 protein (LAG-3), T cell immunoglobulin and mucin 3 domain (TIM-3), T cell immunoreceptor with Ig and ITIM domains (TIGIT), and others, are immunosuppressive molecules, as they negatively regulate activation of the immune effector cells [8]. In cancer, these molecules are deemed responsible for immune exhaustion of the effector cells and downregulation of antitumor response [9]. 

Other checkpoint receptors such as glucocorticoid-induced TNFR-related protein (GITR), CD27, CD40, OX40, and CD137 (4-1BB), which are members of the tumor necrosis factor receptor (TNFR) superfamily, and also checkpoint receptors that belong to the B7-CD28 superfamily, i.e., CD28 itself and inducible T cell co-stimulator (ICOS), are co-stimulating the immune response [10]. Insufficient activity of these molecules in lymphocytes recognizing tumor-related antigens can be one of the causes of the ineffective anticancer immune response. In general, after acquiring the knowledge on the mechanisms of actions of negative and positive immune checkpoints, two main concepts dominate the area of therapies targeting immune checkpoints with monoclonal antibodies. The first is an inhibition (blockade) of negative immune checkpoints with antagonistic antibodies (Figure 1B) and the second is stimulating the positive immune checkpoints with agonistic antibodies (Figure 2B).

## 3. Inhibition of Negative Immune Checkpoints in Cancer

### 3.1. Role of “Classical” Immune Checkpoints—CTLA-4 and PD-L1/PD-1 in Cancer—Early Studies

Discovered in 1987, CTLA-4, is usually referred to as the “classical T cell inhibitory receptor” [11]. The mechanism underlying its immune inhibitory function relies mainly on the competition of CTLA-4 and CD28 in binding to the same ligands—CD80 and CD86 [11]. It is known that high levels of CTLA-4 correlate with reduced activation of T cells primarily in lymph nodes, but also in peripheral tissues and that CTLA-4 expression on T regulatory cells (Tregs) has been shown to be crucial for systemic tolerance [12]. Moreover, it was observed, that *Ctla-4* knockout mice suffer from an expansion of autoreactive and hyperproliferative lymphocytes that eventually take a toll leading to their premature death at the age of 2–3 weeks [13].

Allison et al. have investigated the importance of CTLA-4 signaling in cancer [14]. They revealed that in vivo administration of blocking monoclonal antibodies against CTLA-4 induced tumor rejection and, more importantly, led to the immunity to secondary exposure to tumor cells. This study provided evidence that blockade of CTLA-4 and, therefore, its suppressive activity can enable and potentiate effective immune response against cancer cells in the “brake-off” mechanism [14]. After initial preclinical proof-of-concept studies, in 2000, this strategy was evaluated in patients with advanced cancers. Two fully human CTLA-4–blocking antibodies (ipilimumab and tremelimumab) were used in the first clinical trials [15]. Out of these two antibodies, only ipilimumab received Food and Drug Administration (FDA) approval as the first immune checkpoint inhibitor in cancer treatment in 2011.

Similar to CTLA-4, the role of another “classical” immune checkpoint receptor, i.e., PD-1 in controlling immune tolerance was presented by generating knockout mice [16] by the group of Honjo et al., although the autoimmunity they developed was less severe as compared to CTLA-4 knockout mice. PD-1 expression can be induced on activated B and T cells. Its ligands, programmed death receptor ligand 1 and ligand 2 (PD-L1 and PD-L2), are constitutively expressed at moderate levels in several non-lymphoid tissues, such as heart and lung, with placenta being the most pronounced site for PD-L1 expression [17], but they can also be markedly induced by inflammatory signals in a number of cell types. Thus, the PD-1/PD-L1 axis inhibits T cell activity mostly in the periphery [18].

PD-L1/PD-1 signaling pathway was first linked to tumor immunity in 2002 [19]. Indeed, the overexpression of PD-L1 causes the inhibition of T cell cytolytic activity and thus promoted tumorigenesis, as the effect can be reversed by applying anti-PD-L1 monoclonal antibodies [20]. Several factors can lead to the persistent expression of PD-L1 and/or PD-L2 on tumor cells by, for instance, upregulation by cytokines, chromosomal copy gain [21], disruptions of the PD-L1 3′-untranslated region [22], aberrant activity of pathways mediated by phosphoinositide 3-kinase (PI3K) and protein kinase B (PKB, AKT), epidermal growth factor receptor (EGFR), cyclin-dependent kinase 5 (CDK5), and Janus kinase 2 (JAK2) [21,23], MYC overexpression [24], and viral proteins, e.g., Epstein–Barr virus latent membrane protein 1 (EBV LMP1) [25]. The expression of immunosuppressive PD-L1 molecule can also be induced on other cells presented in the tumor microenvironment (TME), such as endothelial cells, stromal cells, APC, and T cells [26]. Moreover, tumor antigen presentation and TCR triggering are accompanied by interferon-γ (IFN-γ) production, which is a potent stimulator of reactive PD-L1 expression [18]. Therefore, antitumor T cells can be exposed to continuous PD-L1/PD-1 signaling. It causes their exhaustion and inhibits the antitumor cytotoxic T cell response, which can be reversed by anti-PD1/anti-PD-L1 monoclonal antibodies [20].

Currently, the FDA has approved seven monoclonal antibodies targeting classical inhibitory immune checkpoints for the clinical treatment of patients with numerous cancer types: ipilimumab targeting CTLA-4 pathway, and six antibodies targeting PD-L/PD-L1 axis, including atezolizumab, avelumab, durvalumab, nivolumab, cemiplimab, and pembrolizumab. The FDA approval status for each of these antibodies in various cancer types is summarized in Table 2.

### 3.2. Formats of the Anti-Immune Checkpoint Antibodies

The FDA-approved antibodies targeting checkpoints are all the full-size monoclonal antibodies, either human (ipilimubab, nivolumab, avelumab, durvalumab, and cemiplimab) or humanized (pembrolizumab and atezolizumab) of IgG1 or IgG4 subclass (Table 2). All of these antibodies have low or markedly reduced binding to C1q in order to avoid complement-dependent cytotoxicity (CDC) and most of them present reduced binding to Fc receptors in order to diminish the antibody-dependent cellular cytotoxicity (ADCC) (reviewed in [28]). The only exception is avelumab, which is capable of inducing ADCC effect [29]. In experimental settings or early clinical studies, a whole new format of anti-immune checkpoint molecules are the bispecific antibodies (reviewed in [30]), which are described in Section 5 below. Lastly, the antibody-drug conjugate (i.e., anti-PD-L1-doxorubicin) format has also been attempted in preclinical settings [31].

### 3.3. Clinical Application of Anti-CTLA-4, Anti-PD-1 and Anti-PD-L1 Antibodies

Several biomarkers are used to identify patients more likely to respond to CTLA-4 or PD-1/PD-L1 blockade as well as other immunotherapeutics [32]. PD-L1 expression is evaluated as one of them, though it has some limitations. Across all tumor types, patients with PD-L1-negative tumors respond to anti-PD-1/PD-L1 therapy in 0% to 17%, while those with PD-L1-positive tumors exhibit a response rate from 36% to 100% [33]. However, there is no precise definition of what level of PD-L1 expression is to be considered as positive. Additionally, different detection methods are used and, therefore, standardization is limited. Furthermore, nonmalignant cells within the tumor microenvironment (TME) also can express PD-L1. Thus, several other candidate predictive biomarkers were studied, among them, clinical-pathologic factors, gene and phenotypic alterations, tumor microenvironment, and immune effector cells [34]. The total number of mutations per coding area of a tumor genome, i.e., tumor mutational burden (TMB), turned out to be a promising biomarker for immunotherapies [35]. Higher TMB favors positive response to PD-1/PD-L1 blockade in several types of tumors, e.g., non-small cell lung carcinoma (NSCLC) [36].

It is essential to mention that targeting CTLA-4 and the PD-L1/PD-1 axis simultaneously in cancer patients has produced synergistic effects in numerous cases. Therefore, combinations of ipilimumab with, for instance, nivolumab, have been approved in several types of cancer [37].

### 3.4. Recent Developments in the Application of Anti-CTLA-4 Antibodies

There is also an ongoing study (NCT03860272) on an improved version of the anti-CTLA-4 monoclonal antibody, AGEN1181, that harbors an engineered Fc domain that increases the stability and half-life of the antibody. This Phase 1 study enrolls patients with refractory, advanced cancer (solid tumors) regardless of diagnosis and prior therapies.

One of the studies has also reported an anti-CTLA-4 antibody that is preferentially released within the tumor microenvironment. Specifically, the CTLA-4 dual variable domain Ig (anti-CTLA-4 DVD) was designed to have the inner CTLA-4-binding domain shielded by an outer tumor-targeting anti-prostate stem cell antigen (PSCA) domain. Once cleaved in the TME, the shield exposes the inner CTLA-4-binding site. The targeted release of anti-CTLA-4 within tumors was found to deplete tumor Tregs, without affecting tissue-resident Tregs, thus not inhibiting their antitumor activities [38].

### 3.5. Adverse Effects of Immune Checkpoint Inhibition

The grand proportion of patients treated with immune checkpoint inhibitors have experienced drug-induced immune-related adverse events (irAEs). The toxicity of the anti-CTLA-4 antibody was low in most cases, but some patients also experienced severe and life-threatening irAEs [39]. Generally, irAEs are more likely to appear in patients treated with anti-CTLA-4 (60–85%) than anti-PD-1 (16–37%), or anti-PD-L1 (12–24%). However, when the combination of anti-CTLA-4 and anti-PD-1 or anti-PD-L1 was applied, the frequency and severity of irAEs were higher compared to single-agent treatment—up to 60% of patients on combined therapy develop severe side effects that can include autoimmune inflammation in the nervous system [40] and heart [41]. Some studies reported that the frequency may be even higher and appear in even 91% of patients [42]. The most common irAEs include rash, colitis, hepatitis, endocrinopathies, and pneumonitis [43]. Neutrophilic dermatoses are another often an adverse effect of checkpoint inhibitors [44,45]. The management of checkpoint inhibition-induced irAE usually bases on immunosuppression with corticosteroids or other immunosuppressant agents [43].

### 3.6. Combination Therapies Using Checkpoint Inhibitors

In general, the fundamental problem with the application of immune checkpoint inhibitors is that the presence of T cells in the tumor site is a limiting factor. The lack of effector T cells within the tumor borders makes the elimination of cancer cells via the immune “brake-off” mechanism nearly impossible [46]. Another issue related to the adaptation of cancer cells is checkpoint inhibition by amplification of other negative checkpoint molecules in order to keep the exhausted phenotype of tumor-infiltrating lymphocytes (TILs). The latter phenomenon is being addressed by the application of antibodies inhibiting additional negative checkpoints, as discussed further on.

The problem of initially non-existent tumor infiltrates can be solved by increasing the immunogenicity of the tumor cells, e.g., by induction of the immunogenic cell death by cytotoxic or immuno-stimulatory approaches, or by using the molecularly targeted therapies. Indeed, in order to improve the safety and efficiency of therapies based on immune checkpoint blockade, several combinations are now tested both with non-biological and biological agents. The examples are combined interventions with non-biological approaches are surgery [47], radiation therapy [48], chemotherapy [49], and potentially targeted therapies as well [50]. There is also a substantial number of biological agents for combinatory treatment with immune checkpoint inhibitors either in preclinical or clinical settings, e.g., other therapeutic monoclonal antibodies (NCT02914405), therapeutic vaccines [51], natural or synthetic cytokines [52], anti-cytokine antibodies (NCT03111992), oncolytic virotherapy [53], or immune effector cells used in adoptive therapies [54]. The last one is especially interesting, as the use of lymphocytes guided with natural or synthetic tumor antigen-specific receptors can overcome the problem of non-existent immune cell infiltrates in the tumor. Conversely, as the surrounding microenvironment of solid tumors can use negative checkpoint molecules to hamper the effectiveness of CAR (chimeric antigen receptor)-T-based therapies, the addition of checkpoint blockade can significantly improve the efficacy of adoptive approaches [55,56]. Indeed, several clinical trials of this manner are being currently conducted (e.g., NCT03726515, NCT04003649).

### 3.7. Antibodies Against Novel Negative Checkpoints

Due to several limitations that appear in therapies based on the use of the anti-CTLA-4, PD-1, or PD-L1 monoclonal antibodies, both as monotherapy or in combination regimens, the additional co-inhibitory pathways are intensively investigated as novel pharmacological targets. Next-generation monoclonal antibodies targeting alternative immune checkpoints in the tumor microenvironment are being explored in clinical trials (reviewed in [57]). The overview of the new target candidates for immune checkpoint inhibition along with the specific antibodies and example clinical trials are presented in Table 3.

LAG-3 is a negative checkpoint receptor that effectively suppresses T cells activation and cytokines secretion, thereby maintaining immune homeostasis [58]. LAG-3 is expressed primarily on activated effector T cells and Tregs, but also other types of immune cells. The precise molecular mechanisms for inhibitory LAG-3 signaling remain unclear. MHC II is considered to be a canonical ligand for LAG-3, however there is a lack of direct evidence for the protein–protein interaction between these two molecules. Recent studies have reported that fibrinogen-like protein 1 (FGL1) may be a major functional ligand of LAG-3, especially on target cells. The expression levels of both FGL1 mRNA and protein are limited to the liver and pancreas in normal conditions, but has been shown to be upregulated in human solid tumors including lung cancer, prostate cancer, melanoma, and colorectal cancer compared to normal tissues [59]. What is apparent, however, is that LAG-3 shows a strong synergy with PD-1 in multiple settings [60]. Indeed, it was observed that dual LAG-3/PD-1 blockade has a much more significant result on resolving the issue of T cell exhaustion, as compared to only LAG-3 blockade. Moreover, tumor growth was more likely to be reduced in both LAG-3- and PD-1-deficient mice, that when the single knockout of one of these molecules was performed [61]. A similar effect was observed in models of colon adenocarcinoma, fibrosarcoma, ovarian tumors, melanomas, lymphomas, and multiple myelomas, where the mice were treated with one antibody or with combination targeting LAG-3/PD-1 compounds. In all these in vivo studies, increased survival and tumor regression appeared mainly due to the restored CD8^+^ T cell function and increased cytokine production. Additionally, the blockade of LAG-3 on CD4^+^ T cells increased their production of interleukin (IL)-2, IL-4, IFN-γ and tumor necrosis factor-alpha (TNF-α) [62]. There are currently several LAG-3-modulating treatments tested in different phases of clinical trials (e.g., NCT03489369, NCT02658981, NCT03311412, NCT03662659). Importantly, the combination of anti-LAG-3 (BMS-986016) and anti-PD-1 (nivolumab) antibodies has been shown to be effective in melanoma patients resistant to anti-PD-1/PD-L1 therapy [63], and is currently being tested in other tumor types (NCT04082364, NCT01968109).

TIGIT is an inhibitory checkpoint receptor that has a role resembling the PD-1/PD-L1-mediated signal in tumor immunity and is upregulated in many types of cancers [64,65]. TIGIT is expressed in both NK cells and T cells (activated, memory, and regulatory) and has a role in their activation and maturation, among others by inducing the generation of mature immuno-regulatory dendritic cells [66]. TIGIT binds as a competitor to the same set of ligands as the CD226 (DNAM-1) receptor: CD155 (poliovirus receptor, PVR) with high affinity and CD112 (Nectin-2 or poliovirus receptor-related 2, PVRL2) with lower affinity [66]. Anti-TIGIT antibodies (e.g., BGB-A1217 and BMS-986207) were shown to act synergistically with inhibition of the anti-PD-1/PD-L1 axis in pre-clinical models [67] and are currently being tested in clinical trials in patients with advanced solid tumors (NCT04047862, NCT02913313).

TIM3 (also known as Hepatitis A Virus Cellular Receptor 2, HAVCR2) also contributes to immune tolerance by providing negative regulation of lymphocyte activation [68]. It is expressed on multiple immune cells, including conventional T cells (activated, memory, and exhausted), Tregs, and innate immune cells [69]. In cancer, chronic stimulation induces TIM3 upregulation in tumor antigen-specific T lymphocytes, especially in CD8^+^ TILs, and, at the same time, peripheral T cells show minimal TIM3 expression. Similar to the PD-1/PD-L1 axis, TIM3 plays a role in T cell exhaustion during chronic immune stimulation [70], and especially in trimming the Th1-type immune responses [69]. Blocking the TIM3 pathway stimulates tumor antigen-specific T cell proliferation and cytotoxic functions, inhibits the activity of Tregs [71], and decreases the presence of myeloid-derived suppressor cells (MDSCs) in tumors [72]. The inhibition of TIM3 by mAbs is currently evaluated in at least 10 ongoing Phase 1 clinical trials, as a single blockade, with combination strategies (as presented in Table 2), or with bispecific antibodies (e.g., NCT03708328 with RO7121661 compound or NCT03752177 with LY3415244).

What is of importance for generation of anti-TIM3 antibodies for immunomodulatory purposes is the fact that at least several different surface molecules have been presented as ligands for TIM3, including galectin-9, phosphatidylserine (PtdSer), high mobility group protein 1 (HMGB1), and perhaps the carcinoembryonic antigen-related cell adhesion molecule 1 (CEACAM1) [73]. These ligands bind to the extracellular portion of the TIM3 molecule at distinct sites. Thus, the question arises which of the TIM-3 epitopes should be targeted by monoclonal antibodies in order to disrupt specific binding of the ligand(s) responsible for the immunosuppressive actions of this receptor. The study by Sabatos-Peyton et al. suggested that targeting the PtdSer and CEACAM1 binding sites is a shared property of anti-TIM-3 antibodies with demonstrated immunomodulatory functions [73]; however, this topic needs further research. An additional argument for the importance of anti-TIM3 antibodies was given by recent studies on a mouse model of lung adenocarcinoma. It was observed that mice and also some patients, who develop adaptive resistance to anti-PD-1 treatment after an initial response, may show a TIM-3 upregulation. Using anti-TIM3 antibodies in these cases can contribute to improved efficacy of the treatment [74].

Targeting negative checkpoints with monoclonal antibodies does not always lead to noticeable beneficial effects in humans, despite promising results in preclinical models, and examples of such developmental paths are V-domain Ig suppressor of T cell activation (VISTA) and CEACAM1 checkpoint receptors, as described below.

VISTA is a membrane receptor constitutively expressed on the immune cells, primarily in myeloid cells, but also detected in T cells and to some extent in NK cells [75]. VISTA presence is more pronounced on the surface of tumor infiltrating myeloid cells and on Tregs within the tumor mass as compared to those in the peripheral tissues [76]. In mouse models, it has been shown that blocking VISTA with monoclonal antibodies decreases MDSCs infiltration in tumors, and in parallel, it increases the presence of immune effector infiltrates. VISTA can also be expressed by tumor cells and induce regulation of T cell function [77] and anti-VISTA antibody had positive effects on the survival of tumor-bearing mice [77]. Therefore, the anti-VISTA antibody (Onvatilimab, JNJ-61610588) was introduced into the Phase 1 clinical trial in 2016 (NCT02671955), but the study was terminated due to the manufacturer’s decision. Despite that, VISTA remains in the focus of cancer studies, as VISTA expressed on intratumor CD68-positive in pancreatic cancer has recently been indicated as an important role player the resistance of these tumors to immune checkpoint inhibitors [78].

CEACAM1 is another recently characterized immune checkpoint. CEACAM1 is expressed at high levels on T cells activated by stimulation with IL-2 or anti-CD3 antibodies or activated NK cells, but also can be expressed on tumor cells and act in homophylic interactions with CEACAM1 on the immune cells [79]. CEACAM1-L, the dominant isoform expressed in most T cells and NK cells, acts as an inhibitory receptor downregulating activation of these cells, e.g., in malignant melanoma [80]. In early preclinical settings, anti-CEACAM1 antibody (CC1) combined with anti-TIM3 intervention generated a robust therapeutic efficacy in mouse intracranial glioma model [81]. Also, CM-24 (MK-6018), a humanized anti-CEACAM1 IgG4 antibody, was demonstrated to increase the cytotoxic activity of lymphocytes against cancer cells in various in vitro and in vivo models [82]. A first clinical trial utilizing CM-24 was initiated in 2015 (NCT02346955) either with CM-24 administered alone or in combination with pembrolizumab, but the study was terminated in 2017, as no efficacy was observed. Subsequently, the anti-mouse CEACAM1 antibody (CC1) has been shown to induce no or minimal antitumor effects in vivo, as a monotherapy or in combination with anti-PD-1 treatment in three mouse models of solid tumors [83]. Interestingly, however, the significant efficacy of another CEACAM1-targeting antibody (MG1124) has recently been reported in combination with pembrolizumab in humanized mice xenografted with human lung cancer cells [84]. The current view is that CEACAM1 plays a multifaceted role as a checkpoint molecule in the human immune system, with either positive or negative effects depending on the circumstances, which might be attributed to the occurrence of various splicing forms of this molecule [85].

## 4. Application of Agonistic Compounds towards Positive Immune Checkpoints in Cancer

The counterbalance for negative immune checkpoints are the stimulatory (positive) checkpoint molecules, also acting mostly in a ligand-receptor manner. Here, the approach in cancer treatment is to use agonistic antibodies that increase signaling from the stimulatory immune checkpoints and thus positively regulate activation of the immune system against cancer (Figure 2). Although, in theory, this is just an opposite intervention as compared to the negative checkpoint blockade, there is a fundamental difference between these two kinds of approaches. Specifically, while the beneficial effects of inhibiting a negative checkpoint can be seen only if this particular checkpoint is used by the tumor to evade the immune system, the activation of a positive immune checkpoint should be stimulating lymphocytes more broadly, regardless of the particular defenses raised by the tumor. This notion is of importance in the stimulatory approach, as it provides broader universality of treatment, but also a higher risk of adverse effects.

The latter phenomenon has been a reason for a spectacular failure of a treatment attempt with an antibody activating the CD28 molecule, the most powerful of the stimulatory immune checkpoints on T lymphocytes. The interaction of CD28 with its cognate ligands, CD80 or CD86, provides the so-called “the second signal” for activation of the T lymphocyte following the recognition by TCR of specific MHC-antigen complex. The agonistic antibody binding to this receptor, theralizumab (also known as TGN1412, CD28-SuperMAB, or TAB08) was initially designed to treat B cell chronic lymphocytic leukemia (B-CLL) [86]. It was found safe in preclinical studies and, therefore, applied to six trial participants in Phase 1 clinical trial in 2006. Unexpectedly, in all cases, theralizumab induced a severe cytokine release syndrome with a high proportion of multiple organ failure [87]. This incident caused the temporary withdrawal of this anti-CD28 agonistic antibody from further studies in humans and led to a revision of European guidelines for first-in-man phase-1 clinical trials for biologic agents [88]. Despite the initial failure of theralizumab, the Phase 1 trial in patients with advanced neoplasms was initiated in December 2016 (NCT03006029) and is ongoing.

Other positive immune checkpoints are also seen as promising targets in anticancer treatment. The list of the clinical trials with the respective agonistic compound are presented in Table 2. The main difference between CD28 and other stimulatory receptors is that CD28 is present on naïve T cells, while the rest are being expressed mostly following stimulation of the lymphocytes. This increases the safety level of agonistic antibodies against such receptors as OX40, CD137, GITR, ICOS, or CD27.

OX40 (CD134, TNFRSF4) belongs to the superfamily of TNFR and can be detected on the surface of activated CD4^+^ and CD8^+^ T cells, and also on Tregs, NK cells, and neutrophils. The expression of its natural ligand, OX40L (CD52), can be induced by proinflammatory cytokines on dendritic cells (DCs), macrophages, B lymphocytes, and also endothelial or smooth muscle cells. OX40 is detected on lymphocytes 24 to 72 h after activation [89]. Stimulation via OX40 has been shown to overcome the negative effects induced by CTLA-4 in T lymphocytes and also to antagonize the suppressive effects of Tregs on the activation of the effector cells [90]. Thus, agonistic antibodies or Fc fusion proteins against OX40, such as BMS986178, GSK3174998, PF-04518600, MEDI6383, MEDI6469, or MOXR0916, have been generated for use against a range of malignancies (reviewed in [90]). Generally, these antibodies are well tolerated in cancer patients and have a mild toxicity profile. Interestingly, the application of anti-OX40 stimulatory compounds (MEDI6383 or MEDI6469) was shown to induce activation and proliferation of T cell populations in cancer patients [91], but also resulted in upregulation of PD-L1 in tumor cells, occurring between 12 and 19 days following the infusion (NCT02274155). This indicates the potential synergism of agonistic anti-OX40 compounds with anti-PD-1/PD-L1 blocking antibodies. Indeed, such combinations, e.g., PF-04518600 with avelumab (NCT03971409) or GSK3174998 with pembrolizumab (NCT02528357), are being tested in clinical trials. In recent preclinical studies, spectacular effects against a range of malignancies have been reported in the combination of anti-OX40 agonist (BMS 986178) with TLR9 agonist SD-101 [92]. This combination is currently tested in Phase 1 clinical trial in advanced cancers (NCT03831295).

CD137 (4-1BB, TNFRSF9) can be primarily detected on activated CD8^+^ and CD4^+^ T cells but following induction with proinflammatory stimuli also appears on other cell types, including NK cells, B cells and dendritic cells, or endothelial cells following induction with pro-inflammatory stimuli [93]. CD137 ligand (4-1BBL, CD252) is expressed on various antigen-presenting cells. Ligation of CD137 results in a pro-stimulatory signal, enhancing among others the tumor-selective cytotoxicity of CD8^+^ T lymphocytes and NK cells and secretion of IFN-γ [94]. The examples of agonistic anti-CD137 antibodies are utomilumab (PF-05082566) and urelumab (BMS-663513). These antibodies are being assessed in numerous clinical trials in cancer patients, mainly in combinations with other immunomodulatory compounds. Notably, urelumab, as an agonist for CD137, appears to act with higher potency than utomilumab [95], which is attributed to different epitopes bound by these antibodies on CD137 molecule [96]. Therefore, a somewhat higher frequency of adverse effects has been reported with urelumab. Similarly to OX-40, anti-CD137 antibodies act in synergy with the inhibitors of PD-1/PD-L1 axis [95], and such combinations are being evaluated in clinical trials. In line with this tendency, a recombinant PD-L1/4-1BB bispecific antibody, ES101, has recently been developed and subjected to Phase 1 clinical trial in advanced solid malignancies (NCT04009460).

GITR (TNFRSF18), also known as activation-inducible tumor necrosis factor receptor (AITR), is another member of the TNFR family, the expression of which is increased upon activation on T and NK cells, and also the CD4^+^CD25^+^ regulatory T cells. GITR ligand (GIRTL) is primarily expressed on antigen-presenting cells and endothelium. Stimulation of GITR has been shown to increase the effector activity of T cells and decrease the immunosuppressive effects elicited by Tregs. Numerous agonistic anti-GITR antibodies have been developed, for instance BMS-986156, TRX-518, AMG 228, INCAGN01876, MEDI1873, MK-4166, and GWN323, all of which are currently being tested in clinical trials in solid or hematological malignancies. Generally, the application of these antibodies is considered safe, with only relatively mild adverse effects [97]. However, most of these antibodies produce quite modest immunostimulatory effects or clinical response rates so far [97]. That indicates that anti-GITR antibodies may be better candidates for combined approaches rather than to be used in monotherapy [98].

ICOS (CD278), unlike most of the costimulatory receptors on T cells, belongs to the superfamily of CD28-type molecules. ICOS is expressed at low levels on naïve T cells, but its expression is significantly increased following activating stimuli and plays a role in T cell activation and governing Th1-, Th2-, and Th17-type responses [99]. The ligand of ICOS (ICOSLG, B7-H2, CD275) appears mostly on APC, including B cells, dendritic cells, and macrophages, and also on non-immune cells following stimulation with lipopolysaccharide [99]. ICOS is also considered a target for agonistic approach in anticancer therapies [100], and therefore several agonistic (such as JTX-2011, GSK3359609, BMS-986226) antibodies were generated, but also an antagonistic (MEDI-570) antibody, as well. Interestingly, the ICOS–ICOSL pathway can sometimes exhibit pro-tumorigenic effects due to the inducing generation and function of Tregs [101], and in particular cases, an inhibitory approach to ICOS signaling might be of preference [100].

CD27 is another member of the TNFR superfamily. Its ligand, CD70, is expressed on highly activated lymphocytes, but also in T- and B-cell lymphomas [102]. Varlilumab (CDX-1127) is an anti-CD27 agonistic antibody that has been and is being evaluated in several clinical trials in solid malignancies (e.g., NCT04081688, NCT02335918). An interesting fact is that CD70 is currently under investigation as a target for the ARGX-110 antibody in the treatment of hematological malignancies (NCT03030612), and the previous study in solid tumors (NCT02759250) has shown promising signs of its biological activity.

## 5. Perspectives in Immune Checkpoint Targeting—Bispecific Antibodies

The most significant challenge of immunomodulation is balancing between the potency of antitumor effects and the severity of autoimmune and/or inflammatory adverse events. The potential solution for this problem may rely on choosing the appropriate combination of immunomodulatory compounds and potentially other anticancer modalities in order to tip this balance towards the antitumor effectiveness. A derivative of such an approach is using multivalent (e.g., bispecific) antibodies, that combine the beneficial effects of multiple checkpoint targets.

Bispecific antibodies (bsAb) are engineered antibody-derived fusion proteins designed to bind two epitopes (usually on two different antigens) simultaneously. The global development of bsAb is thriving. Currently, there are numerous (>20) technology platforms commercialized for bsAb formation and development, and more than 85 bsAb are in clinical development with two of them already marketed [103]. The most pronounced advantage of bsAb over the combinations of full-size format mAb is that bsAb can bring their targets into close proximity. Also, the amount of balance between both binding arms of bsAb is always 1:1 at the site of destination, while for combinations of full-size mAb, it can be severely shifted in either way depending on circumstances. From the perspective of immune checkpoint targeting, three functional formats of bsAb are applicable [30]:

- Redirectors of cytotoxic effector cells—these bsAb bind to the tumor-associated antigen (a checkpoint molecule in this case) and the molecule responsible for activation of the effector cells (e.g., CD3 on T/NKT cells or CD16 on NK/NKT cells). Such bsAb are also referred to as bi-specific T cell engager (BiTE) or bi-specific killer cell engager (BiKE);
- Dual immunomodulators—the principle of action of these bsAb is to bind two checkpoint molecules simultaneously, usually on the same cell.
- Tumor-associated antigen-targeted immunomodulators—these bsAb bind to the tumor-associated antigen (on cancer cell) and a checkpoint molecule (e.g., a positive immune checkpoint on the effector cell). The difference between these bsAb and redirectors of cytotoxic effector cells is that they would not discriminate between the effector cell type, as long as the positive immune checkpoint molecule is expressed.

The examples of bsAb at various phases of preclinical or early clinical development are presented in Table 4.

## 6. Summary and Future Directions

Multiple lines of evidence suggest that modulation of the intrinsic antitumor response by using antagonistic compounds towards negative immune checkpoints and agonistic factors stimulating positive immune checkpoints is a highly promising strategy in cancer treatment. The chemical assembly of such immunomodulatory agents is most often based on antibody structure or an antibody derivative. Numerous cancer patients have already achieved long-term remissions from malignancies, that would be lethal prior to the immunotherapy era. Nevertheless, the immunomodulatory approach to cancer treatment is far from perfect. Another solution might be an attempt to mimic as close as possible the natural course of activation of T cells, which should be localized and limited to the site(s) of disease rather than systemic. This effect can be potentially achieved by the intratumoral application of a given immunomodulator with hope for inducing an abscopal effect against the remaining tumors. Also, the identification of new antibody-targetable immune checkpoints can allow for a higher percentage of objective responses in cancer patients resistant to current therapies. An ideal expression profile is high and homogeneous antigen expression on the surface of all tumor cells and absent from normal tissue. However, some types of cancers are made up of multiple cancer subtypes that evolve with time. The recently used approaches to solve this problem are using transcriptome analysis of cancer cells [105,106], also integrated with cell-surface proteomic data [107]. Another novel approach is to identify tumor antigens by analyzing antibodies derived from local lymph nodes [108], which could allow one to also identify tumor antigens expressed by individual cancer patients.

Lastly, elaboration of more precise criteria for evaluation immune-modified responses in solid tumors along with properly chosen companion diagnostics would allow for better identification of patients benefiting from the immunomodulatory therapies. Despite the remaining challenges, the application of antibody-based modulators of immune checkpoints has already opened a new pathway for personalized and precise treatment strategies in cancers and is undoubtedly one of the most significant breakthroughs in current medicine.

## Figures and Tables

**Table 1:** Examples of suppressive (negative) and stimulatory (positive) immune checkpoint ligand–receptor pairs with cellular distribution of these molecules under physiological conditions.

Ligand	Cellular Distribution of the Ligand Expression	Immune Checkpoint Receptor	Cellular Expression of the Receptor Expression
Suppressive (negative) immune checkpoints			
CD80 or CD86	Antigen-presenting cells	CTLA4	Activated T cells, Tregs
PD-L1 (CD274) or PD-L2 (CD273)	DCs, macrophages, peripheral non-lymphoid tissues	PD-1	Activated B and T cells, APCs, NK cells
MHC class II/Lectins	Antigen-presenting cells	LAG3	Activated T cells, Tregs, NK cells, B cells, DCs
CD155/CD112	Normal epithelial, endothelial, neuronal, and fibroblastic cells	TIGIT	Activated T cells, Tregs, NK cells
Galectin 9/ PtdSer /HMGB1	Multiple tissues	TIM3	Activated T cells
VSIG-3	Neurons and glial cells	VISTA	Naïve and activated T cells
CEACAM1	T and NK cells	CEACAM1	Activated T and NK cells
Stimulatory (positive) immune checkpoints			
B7 molecules: CD80 or CD86	Antigen-presenting cells	CD28	T cells
OX40L	DCs, macrophages, B cells, endothelial cells, smooth muscle cells	OX40	Activated T cells, Tregs, NK cells, neutrophils
CD137L	Antigen-presenting cells	CD137 (4-1BB)	Activated Tcells, NK cells, B cells, DCs, endothelial cells
GITRL	Antigen-presenting cells and endothelium	GITR	T and NK cells, Tregs
ICOSLG	APCs, B cells, DCs and macrophages	ICOS	Naïve and activated T cells
CD70	Activated lymphocytes	CD27	Activated T and NK cells

**Table 2:** The list of Food and Drug Administration (FDA)-approved monoclonal antibodies acting as inhibitors of negative checkpoints in human cancer [27].

Checkpoint Inhibitor	Antibody Format	Examples of Types of Cancers with FDA-Approved Use	Year of First Approval
Ipilimumab	Human anti-CTLA4 IgG1	Melanoma, renal cell carcinoma, metastatic colorectal cancer	2011
Pembrolizumab	Humanized anti-PD-1 IgG4	Melanoma, non-small-cell lung cancer, renal cell carcinoma, urothelial bladder cancer, Hodgkin lymphoma, head and neck cancer, Merkel cell carcinoma, microsatellite instability-high cancer, gastric cancer, hepatocellular carcinoma, cervical cancer, primary mediastinal large B-cell lymphoma	2014
Nivolumab	Human anti-PD-1 IgG4	Melanoma, non-small-cell lung cancer, renal cell carcinoma, urothelial bladder cancer, Hodgkin lymphoma, head and neck cancer, colorectal cancer, hepatocellular carcinoma, small cell lung cancer	2014
Atezolizumab	Humanized anti-PD-L1 IgG1	Non-small-cell lung cancer, urothelial bladder cancer, small cell lung cancer, breast cancer	2016
Avelumab	Human anti-PD-L1 IgG1	Merkel cell carcinoma, urothelial bladder cancer	2017
Durvalumab	Human anti-PD-L1 IgG1	Non-small-cell lung cancer, urothelial bladder cancer	2017
Cemiplimab	Human anti-PD-1 IgG4	Cutaneous squamous-cell carcinoma	2018

**Table 3:** Inhibitory and stimulatory immune checkpoint molecules with respective anti-receptor antagonistic antibodies and examples of clinical trials.

Receptor	Antagonistic Compounds	Example Clinical Trials (Phase)	Comments
Inhibitory immune checkpoint molecules			
LAG-3	MGD013 (Anti-PD-1, anti-LAG-3 dual checkpoint inhibitor)	NCT04082364 (Phase 2/3)	HER2-positive gastric cancer or gastroesophageal junction cancer to determine the efficacy of margetuximab combined with anti-HER2 monoclonal antibody and margetuximab combined with anti-HER2 monoclonal antibody or MGD013 and chemotherapy compared to trastuzumab combined with chemotherapy (Cohort B)
	Relatlimab (BMS-986016)	NCT01968109 (Phase 1)	Administered alone and in combination with nivolumab in patients with solid tumors: non-small cell lung cancer, gastric cancer, hepatocellular carcinoma, renal cell carcinoma, bladder cancer, squamous cell carcinoma of the head and neck, and melanoma.
TIGIT	BGB-A1217	NCT04047862 (Phase 2)	Evaluation of anti-tumor effect of BGB-A1217 in combination with tislelizumab in patients with advanced solid tumors.
	BMS-986207	NCT02913313 (Phase 1/2a)	Advanced or spread solid cancers. Administered alone and in combination with nivolumab
TIM-3 (HAVcr2)	Sym023	NCT03489343 (Phase 1)	As a monotherapy in patients with locally advanced/unresectable or metastatic solid tumor malignancies or lymphomas
	TSR-022	NCT02817633 (Phase 1)	As a monotherapy and in combination with an anti-PD-1 antibody and/or an anti-LAG-3 antibody, in patients with advanced solid tumors
	MBG453	NCT03961971 (Phase 1)	MBG453 with stereotactic radiosurgery and spartalizumab in treating patients with recurrent glioblastoma multiforme
		NCT03066648 (Phase 1)	As a monotherapy and in combination with an anti-PD-1 antibody (PDR001) and/or Decitabine in acute myeloid leukemia and high risk myelodysplastic syndromes patients
VISTA	JNJ-61610588	NCT02671955 (Phase 1)	Evaluation the safety and tolerability of JNJ-61610588 in participants with advanced cancer—study terminated.
CEACAM1	CM-24 (MK-6018)	NCT02346955	Advanced or recurrent malignancies, administered as monotherapy or in combination with pembrolizumab—study terminated.
Stimulatory immune checkpoint molecules			
CD28	Theralizumab (TAB08)	NCT03006029 (Phase 1)	Metastatic or unresectable advanced solid malignancies
OX40 (CD134)	BMS 986178	NCT03831295 (Phase 1)	Advanced solid malignancies, combination with TLR9 agonist SD-101
	MEDI6469	NCT02274155 (Phase 1)	Head and neck squamous cell carcinoma
	PF-04518600	NCT03971409 (Phase 2)	Triple negative breast cancer, combination with nivolumab
	GSK3174998	NCT02528357 (Phase 1)	Advanced solid tumors, combination with pembrolizumab
	MOXR0916	NCT02219724 (Phase 1)	Locally advanced or metastatic solid tumors
4-1BB (CD137)	Utomilumab (PF-05082566)	NCT03364348 (Phase 1)	Advanced HER2-positive breast cancer, combination with trastuzumab
		NCT02179918 (Phase 1)	Advanced solid tumors, combination with PD-1 inhibitor MK-3475
	Urelumab (BMS-663513) ES101	NCT02534506 (Phase 1)	Advanced malignancies, alone or in combination with nivolumab
		NCT04009460 (Phase 1)	Advanced solid tumors, anti-PD-L1/4-1BB bispecific antibody
GITRL	BMS-986156	NCT02598960 (Phase 1/2)	Advanced solid tumors, alone or with nivolumab
	TRX-518	NCT01239134 (Phase 1)	Solid malignancies
		NCT02628574 (Phase 1)	Advanced solid tumors, in combination with gemcitabine, pembrolizumab, or nivolumab
	AMG 228	NCT02437916 (Phase 1)	Advanced solid tumors
ICOSLG, (CD275)	JTX-2011	NCT02904226 (Phase 1/2)	Advanced solid malignancies, alone or in combination with nivolumab
	GSK3359609	NCT02723955 (Phase 1)	Advanced solid tumors, alone or in combination with pembrolizumab
	BMS-986226	NCT03251924 (Phase 1/2)	Advanced solid tumors, alone or in combination with nivolumab or ipilimumab
	MEDI-570	NCT02520791 (Phase 1)	T-cell lymphomas, antagonistic antibody
CD27	Varlilumab (CDX-1127)	NCT04081688 (Phase 1) NCT02335918 (Phase 1/2)	Non-small cell lung carcinoma, combination with atezolizumab and radiation therapy Five types of solid tumors, combination with nivolumab

**Table 4:** Examples of bispecific antibodies targeted against immune checkpoint molecules.

Antigens	Name	Cancer Type	Reference or Clinical Trial No.
Redirectors of cytotoxic effector cells			
Anti-PD-L1/CD3		PD-L1-positive human cancers	Preclinical [104]
Dual immunomodulators			
Anti-PD-1/TIM3	LY3415244	Advanced solid tumors	NCT03752177 (Phase 1)
Anti-PD-1/TIM3	RO7121661	Metastatic Melanoma Non-small Cell Lung Cancer (NSCLC) Small Cell Lung Cancer (SCLC)	NCT03708328 (Phase 1)
Anti-PD-1/PD-L1	LY3434172	Advanced solid tumors	NCT0393695 9(Phase 1)
Anti-PD-1/CTLA-4	AK104	Gastric Adenocarcinoma Advanced Solid Tumors Gastroesophageal Junction Adenocarcinoma	NCT03852251 (Phase 1/2)
		Advanced Cancer	NCT03261011 (Phase 1)
Anti-CTLA-4/OX40	ATOR-1015	Advanced and/or Refractory Solid Malignancies	NCT03782467 (Phase 1)
Anti-LAG-3/PD-L1	FS118	Advanced Cancer Metastatic Cancer	NCT03440437 (Phase 1)
Tumor-associated antigen-targeted immunomodulators			
Anti-Her2/4-1BB	PRS343	HER2-positive Solid Tumors	NCT03330561 (Phase 1)
